# Identification of a Prognostic Immune Signature for Esophageal Squamous Cell Carcinoma to Predict Survival and Inflammatory Landscapes

**DOI:** 10.3389/fcell.2020.580005

**Published:** 2020-12-17

**Authors:** Chaoqi Zhang, Yuejun Luo, Zhen Zhang, Zhihui Zhang, Guochao Zhang, Feng Wang, Yun Che, Lingling Fang, Yi Zhang, Nan Sun, Jie He

**Affiliations:** ^1^Department of Thoracic Surgery, National Cancer Center/National Clinical Research Center for Cancer/Cancer Hospital, Chinese Academy of Medical Sciences, Peking Union Medical College, Beijing, China; ^2^Biotherapy Center, The First Affiliated Hospital of Zhengzhou University, Zhengzhou, China

**Keywords:** esophageal squamous cell carcinoma, immune signature, immune checkpoints, inflammatory landscape, individualized medicine

## Abstract

Immunotherapy has achieved success in the treatment of esophageal squamous cell carcinoma (ESCC). However, studies concerning immune phenotypes within the ESCC microenvironment and their relationship with prognostic outcomes are limited. We constructed and validated an individual immune-related risk signature for patients with ESCC. We collected 196 ESCC cases, including 119 samples from our previous public data (GSE53624) to use as a training set and an independent cohort with 77 quantitative real-time polymerase chain reaction (qRT-PCR) data, which we used for validation. Head and neck squamous cell carcinoma (HNSCC) and lung squamous cell carcinoma (LUSC) cohorts were also collected for validation. A least absolute shrinkage and selection operator (LASSO) model and a stepwise Cox proportional hazards regression model were used to construct the immune-specific signature. The potential mechanism and inflammatory landscapes of the signature were explored using bioinformatics and immunofluorescence assay methods. This signature predicted different prognoses in clinical subgroups and the independent cohort, as well as in patients with HNSCC and LUSC. Further exploration revealed that the signature was associated with specific inflammatory activities (activation of macrophages and T-cell signaling transduction). Additionally, high-risk patients exhibited distinctive immune checkpoints panel and higher regulatory T cell and fibroblast infiltration. This signature served as an independent prognostic factor in ESCC. This was the first applicable immune-related risk signature for ESCC. Our results furnished new hints of immune profiling of ESCC, which may provide some clues to further optimize associated cancer immunotherapies.

## Background

As reported by global cancer statistics in 2018, esophageal cancer (EC) is the sixth leading cause of cancer deaths and the seventh most common cancer worldwide (Bray et al., 2018), with an estimated 70% of EC cases occurring in China (Chen et al., 2019; Yin et al., 2020). EC primarily includes two subtypes: esophageal squamous cell carcinoma (ESCC) and esophageal adenocarcinoma (Pennathur et al., 2013). ESCC is the predominant histopathological type in China and accounts for almost 90% of all EC cases (Chen et al., 2019). Despite advancements in the standard treatment of EC, the prospect of enhancing the survival rate for such patients remains dismal (Cohen and Leichman, 2015), with a 5-year overall survival (OS) rate of 15–25% (Pennathur et al., 2013; Chen et al., 2016; Huang and Yu, 2018). Meanwhile, neoadjuvant chemoradiotherapy followed by resection has moderately improved the prognosis of patients with locally advanced ESCC compared to traditional surgery alone (Allum et al., 2011; Shapiro et al., 2015). Owing to their high heterogeneity, patients with ESCC tend to exhibit individual differences in therapeutic efficacy, even under the same clinical guidelines and recommended treatment. This may prevent clinical practices from being able to precisely stratify patients with ESCC, leading to the predicament of depersonalized, and often suboptimal treatment. There is an urgent and obligatory need to search for novel therapeutic strategies and stratification methods for patients with ESCC.

Over past decades, immunotherapy – recognized as a milestone for cancer treatment – has advanced by leaps and bounds and revolutionized available treatment choices for several major cancer types (Akinleye and Rasool, 2019; Liu et al., 2019). A clinical trial study of patients with advanced ESCC found that pembrolizumab (also known as “Keytruda”), acting as a second-line therapy, could remarkably improve OS compared to chemotherapy (Metges et al., 2019). In 2019, the U.S. Food and Drug Administration approved the use of pembrolizumab for patients with advanced ESCC and high PD-L1 expression. Immunotherapy is increasingly important to clinical practice and has emerged as a promising and potentially effective modality for treating ESCC. Several recent studies have focused on immune-related parameters to predict OS in patients with EC, including some important immune molecules and cells. The results of these studies have further indicated the significance of the immune tumor microenvironment (TME) (Huang et al., 2019; Yagi et al., 2019). Unfortunately, precision immunotherapy is hard to achieve without a comprehensive understanding of the TME immune landscape. However, there have been few comprehensive analyses of the immune phenotype within the ESCC microenvironment and its relationship with prognosis and treatment outcomes.

Herein, we sought to establish and validate an immune-related risk signature for patients with ESCC. First, we collected 196 ESCC cases from two independent cohorts consisting of GSE53624 and 77 frozen tumor samples. Then, we constructed a risk signature by profiling an immune-related gene set with information extracting from the GSE53624 cohort. This signature was later validated in the independent cohort. We subsequently built a practicable signature that was able to identify high-risk patients with ESCC. These patients generally exhibit worse survival than low-risk patients, both effectively and accurately. Such a signature would be useful for the clinical management and stratification of patients and will also help us understand the association between the ESCC immune TME and corresponding prognostic outcomes.

## Materials and Methods

### Public mRNA Data and Samples Collection

We used 196 ESCC cases in the present study, including 119 samples from our previously reported public data and 77 frozen surgically resected ESCC tissue samples from an independent cohort. We also downloaded a total of 1011 lung squamous cell carcinoma (LUSC) and HNSC samples from The Cancer Genome Atlas (*TCGA*) database^1^.

The correlative mRNA expression data and corresponding clinical information of 119 ESCC samples are publicly available (GSE53624) (Li et al., 2014). We also matched the unpublished recurrence-free survival (RFS) data with these 119 patients. The mRNA expression data of GSE53624 were log2 transformed and quantile normalized, and the mean expression was regarded as the expression of genes with several probes. The 77 frozen tumor tissues collected from the First Affiliated Hospital of Zhengzhou University from 2011 to 2014. This research was approved by the Ethics Committee Board of the First Affiliated Hospital of Zhengzhou University.

### Quantitative Real-Time Polymerase Chain Reaction Analysis

The quantitative real-time polymerase chain reaction analysis (qRT-PCR) analysis assessed the expression of immune-related genes in ESCC samples. Both RNA extraction and cDNA synthesis were based on the manufacturer’s protocol. We employed a 10 μL volume system, which includes 5 μL SYBR Green Master Mix (Invitrogen), 3 μL nuclease-free water, 1 μL template, and 1 μL of each PCR primer in the Agilent Mx3005P Real-Time PCR system. After that, all cDNA samples were diluted for qRT-PCR analysis. The expression values of six target genes were normalized to GAPDH, and then log2 transformed for the next analysis. The primer sequences of the six target genes and GAPDH used for qRT-PCR were displayed in Supplementary Table 1.

### Immunofluorescence Technique

Esophageal squamous cell carcinoma tissues were fixed by using 10% neutral-buffered formalin and embedded in paraffin. Then the tissue sections (3 μm) underwent deparaffinization and blocking for subsequent experiments. Phosphate-buffered saline (PBS), including 2% bovine serum albumin, was used to dilute the primary and secondary antibodies, which were applied to stain α-SMA and Foxp3. Next, the cells were washed three times by PBS and staining the cell nuclei using 4,6-diamidino-2-phenylindole (DAPI). Two independent experiments were performed.

### Functional Enrichment Analysis

Gene Ontology (GO) and Kyoto Encyclopedia of Genes and Genomes (KEGG) pathway analyses were carried out in DAVID 6.8^2^ and Cytoscape 3.7.2^3^.

### xCell and Gene Set Variation Analysis

xCell^4^ is a novel tool used to analyze the cellular heterogeneity landscape through gene profiles in bulk tumors including almost 64 different immune and stromal cell types (Aran et al., 2017). It was used to estimate the abundance of immune and stromal cell type of each patient with ESCC. Also, gene set variation analysis (GSVA) was performed with the *GSVA* package included with R software version 3.5.1.

### Signature Generation and Statistical Analysis

A univariate Cox proportional regression analysis was used to screen the immune-related genes notably associated with OS. Then, we used a least absolute shrinkage and selection operator (LASSO) model to determine which prognostic genes exhibited one standard error (SE) of the minimum criteria. Finally, considering the expression of selected genes and correlation estimated Cox regression coefficients, a risk score formula was generated for each patient. The patients were divided into high- and low-risk groups based on the optimal cutoff point, which was determined by the “surv_cutpoint” function of the “survminer” R package. OS of high- and low-risk patients was calculated using the Kaplan–Meier survival analysis method. The univariate and multivariate Cox proportional hazards regression model was performed to identify whether the risk score was an independent prognostic factor. All data analyses and generation of figures were achieved by R software version 3.5.1^5^ and SPSS 25.0 software. All statistical tests were two-sided. *P* < 0.05 was regarded as statistically significant.

## Results

### Immune-Related Profiles Display Significant Differences Between ESCC Tissues and Adjacent Normal Tissues

**FIGURE 1:**
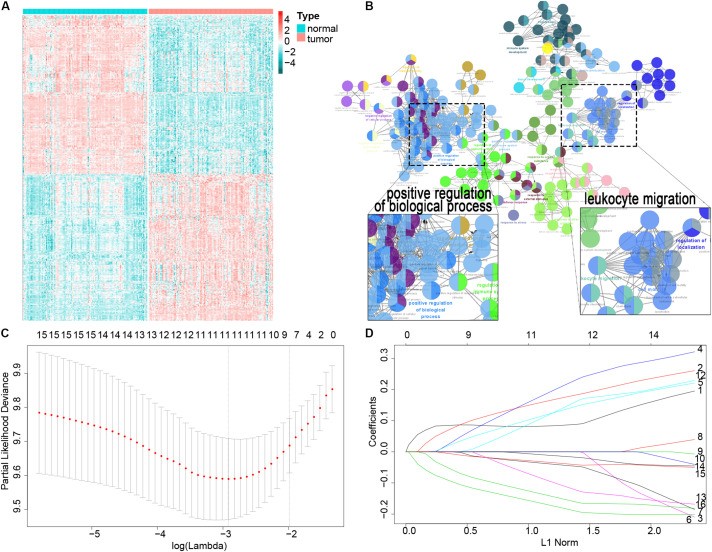
Filter out the most prognostic candidates from the differentially expressed immune-related genes in ESCC. **(A)** Heatmap of differentially expressed immune-related genes between ESCC and adjacent normal tissues. **(B)** GO analysis in Cytoscape of these differential genes. **(C)** One-hundred-fold cross-validation for tuning parameter selection in the LASSO model. **(D)** LASSO coefficient profiles of the most useful prognostic genes.

A total of 119 patients with ESCC with clinical data from GSE53624 were included as the training cohort, and the demographics of the cohort are listed in Supplementary Table 2. We downloaded 3,104 immune-related genes from the AmiGO2 website, and finally, 2,630 genes were matched in the GSE53624 training cohort. We analyzed the matched genes expression in ESCC tissues versus adjacent tissues. Among those immune-related genes, 513 were differentially expressed in ESCC and adjacent tissues (*P* < 0.001) (Figure 1A). GO analysis using Cytoscape 3.7.2 was performed to clarify the biological processes and pathways of these significant genes, which were mostly involved in the positive regulation of biological processes and leukocyte migration (e.g., intracellular signal transduction, cellular protein metabolic process, cell migration, and motility) (Figure 1B).

### Construction of the Immune-Related Prognostic Signature

First, the univariate Cox proportional regression analysis showed that 16 immune-related genes were statistically associated with OS (*P* < 0.01) (Supplementary Table 3). We used the LASSO Cox regression model to filter the immune-related genes with the most prognostic value, and one SE of the minimum criteria was chosen. Eight genes were selected by this procedure: *TSPAN2*, *AMBP*, *ITLN1*, *C6*, *PRLR*, *RBM47*, *PLA2GS*, and *MADCAM1* (Figures 1C,D). Then, to optimize this model and reduce variables, a stepwise Cox proportional hazards regression analysis was performed. This method filtered out a six-gene (*TSPAN2*, *AMBP*, *ITLN1*, *C6*, *PRLR*, and *MADCAM1*) prognostic model. To clearly reveal the screening process of these six genes, a pipeline is presented as Supplementary Figure 1.

**FIGURE 2:**
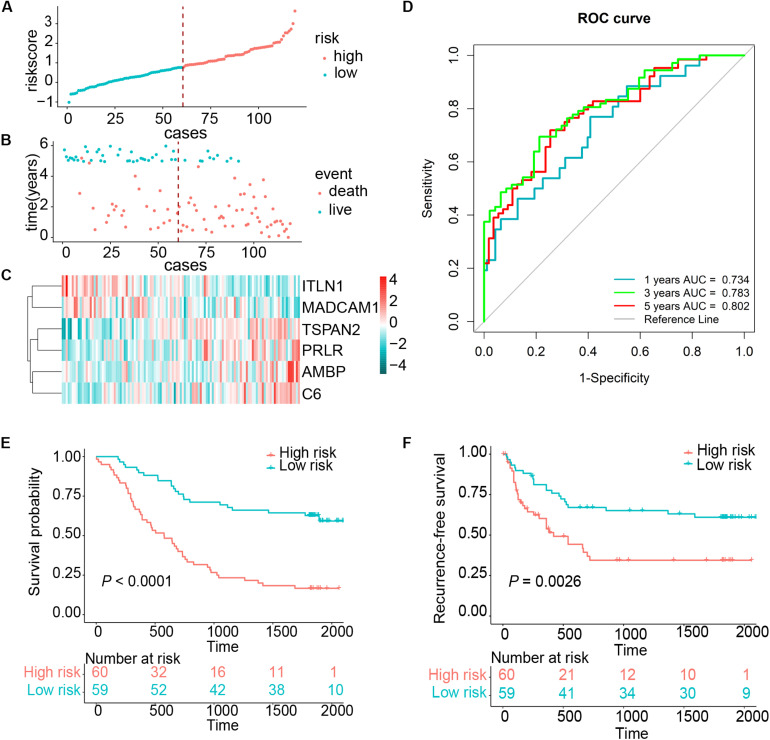
Risk score distribution and survival of patients in the training cohort. **(A)** The risk scores for 119 patients of the training cohort (GSE53624). **(B)** The survival of each patient in the training cohort (GSE53624). **(C)** Expression distribution of the six genes in the training cohort, with red indicating higher expression and blue indicating lower expression. **(D)** ROC analysis of immune-related genes signature for prediction of survival at 1, 3, and 5 years in the training cohort. **(E,F)** Kaplan–Meier curves of OS and RFS in 119 patients of the training cohort based on risk score, respectively.

We established a risk score model based on the expressions of these six genes and corresponding coefficients for patients with ESCC: risk score = (0.1272 × *TSPAN2* expression) + (0.2423 × *AMBP* expression) + (0.2201 × *C6* expression) + (0.1651 × *PRLR* expression) − (0.2720 × *ITLN1* expression) − (0.2724 × *MADCAM1* expression). The risk score of every patient was calculated by this equation. All patients in the training cohort were classified into high- and low-risk groups according to the optimal cutoff point (Figures 2A–C). Patients in the low-risk group had a longer OS than those in the high-risk group [hazard ratio (HR) = 3.7144, 95% confidence interval (CI) = 2.2481–6.1370, *P* < 0.0001] (Figure 2E). Similarly, low-risk patients also exhibited better RFS than high-risk ones (HR = 2.2670, 95% CI = 1.3142–3.9104, *P* = 0.0026) (Figure 2F). The time-dependent area under the receiver operating characteristic (ROC) curves, demonstrating the predictive accuracy of this model, were 0.734, 0.783, and 0.802 in the GSE53624 set at 1, 3, and 5 years, respectively (Figure 2D). To further explore whether the risk score could serve as an independent prognostic factor for ESCC, univariate and multivariate Cox regression analyses were performed in the GSE53624 cohort. After incorporating some important clinical variables, such as age, sex, tobacco use, alcohol use, tumor location, tumor grade, T stage, N stage, and TNM stage, the risk score was still independently related to OS and RFS (Table 1 and Supplementary Table 4).

**TABLE 1:** Univariable and multivariable Cox regression analysis of the six-gene immune-related signature and overall survival in GSE53624 cohort.

	Univariable analysis			Multivariable analysis		
Variable	HR	95% CI	*P* value	HR	95% CI	*P* value
**Age**						
≥60 or <60	1.4206	0.8963–2.2516	0.1352			
**Sex**						
Male or female	1.2094	0.6846–2.1364	0.5126			
**Smoking history**						
Yes or no	0.8596	0.5322–1.3883	0.5361			
Alcohol history	1.0521	0.6559–1.6876	0.8333			
Yes or no						
**Tumor location**						
Upper, middle, or lower	0.9610	0.5827–1.5851	0.8763			
**Tumor grade**						
Well, moderate or poor	1.2195	0.8550–1.7394	0.2733			
**T stage**						
1, 2, 3, or 4	1.1270	0.8387–1.5146	0.4277			
Lymphatic metastasis						
Yes or no	2.1594	1.3191–3.5350	0.0022	1.2331	0.5187–2.9317	0.6354
**TNM stage**						
I, II, or III	1.9011	1.2262–2.9476	0.0041	1.2777	0.5372–3.0389	0.5794
**Risk score**						
High or low	3.7144	2.2481–6.1370	< 0.0001	4.2511	2.4042–7.5166	< 0.0001

*HR, hazard ratio; CI, confidence interval.*

### Validation of the Signature in Stratified Cohorts of ESCC

Because lymph node metastases are important contributors to ESCC prognosis (Kakegawa, 2003), we investigated the relationship between OS and risk score for both lymph node metastasis positive (LN^+^) and lymph node metastasis negative (LN^–^) samples in the GSE53624 cohort. In both subgroups, low-risk patients exhibited significantly longer OS than high-risk ones (Supplementary Figures 2A,B).

In the high-risk group, we also found that this signature suggested significantly poorer OS in the clinical feature subtypes of the training cohort, including early stage, advanced stage, older (age ≥60 years), younger (age <60 years), male, female, non-smoker, smoker, non-drinker, and drinker (Supplementary Figures 3, 4). It is evident that the prognostic performance of the six-gene signature was well validated when the training set was stratified by some important clinical features.

### Validation of the Signature in the Independent Cohort

**FIGURE 3:**
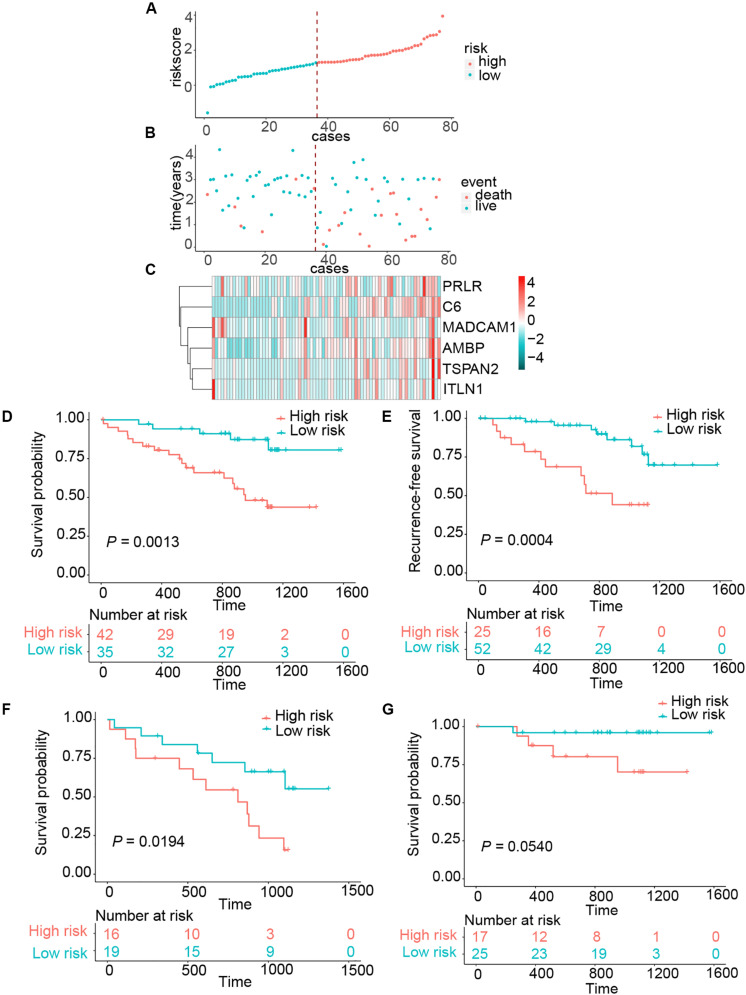
Risk score distribution and survival of patients in the clinical validation cohort. **(A)** The risk scores for 77 patients of the clinical validation cohort. **(B)** The survival of each patient in the clinical validation cohort. **(C)** Expression distribution of the six genes in the clinical validation cohort. **(D,E)** Kaplan–Meier curve of OS and RFS in clinical validation cohort. **(F,G)** Kaplan–Meier curves of OS in LN^+^ and LN^–^ patients of clinical validation cohort.

To assess whether the six-gene signature could be applied in the clinical practice, we further validated in the independent cohort using qRT-PCR analysis. The clinical characteristics of this validation cohort are shown in Supplementary Table 2. Risk scores of all patients were calculated using the same formula and then assigned to high- and low-risk groups accordingly. This risk signature was well validated in the independent cohort. High-risk patients suffered unfavorable prognostic results in OS (HR = 4.4096, 95% CI = 1.6414–11.8465, *P* = 0.0013) and RFS (HR = 4.7875, 95% CI = 1.8440–12.4295, *P* = 0.0004) (Figures 3A–E). In the same way, we detected the connection between OS and risk scores in the LN^+^ and LN^–^ patients, respectively. Patients in the LN^+^ subgroup showed longer OS than their high-risk counterparts. However, this risk score showed a borderline difference between high- and low-risk patients in the LN^–^ subgroup with a *P* = 0.0540 (Figures 3F,G). We also used the same univariate and multivariate Cox regression model to analyze whether the risk score could also function as an independent predictor of prognosis in the independent cohort. As expected, we received the same conclusion as with the training cohort, the risk score was independently associated with OS and RFS (Table 2 and Supplementary Table 5).

**TABLE 2:** Univariable and multivariable Cox regression analysis of the six-gene immune-related signature and overall survival in the independent validation cohort.

	Univariable analysis			Multivariable analysis		
Variable	HR	95% CI	*P* value	HR	95% CI	*P* value
**Age**						
≥60 or <60	2.2471	0.7655–6.5952	0.1406			
**Sex**						
Male or female	1.5951	0.5438–4.6793	0.3951			
Lymphatic metastasis						
Yes or no	5.3376	1.9912–14.3080	0.0009	2.9974	0.9399–9.5586	0.0636
**TNM stage**						
I, II or III	2.0551	1.2530–3.3709	0.0043	1.5058	0.7484–3.0298	0.2512
**Risk score**						
High or low	4.4097	1.6414–11.8465	0.0033	2.8332	1.0113–7.9372	0.0475

*HR, hazard ratio; CI, confidence interval.*

### Biological Pathways Analysis of the Immune-Related Signature

**FIGURE 4:**
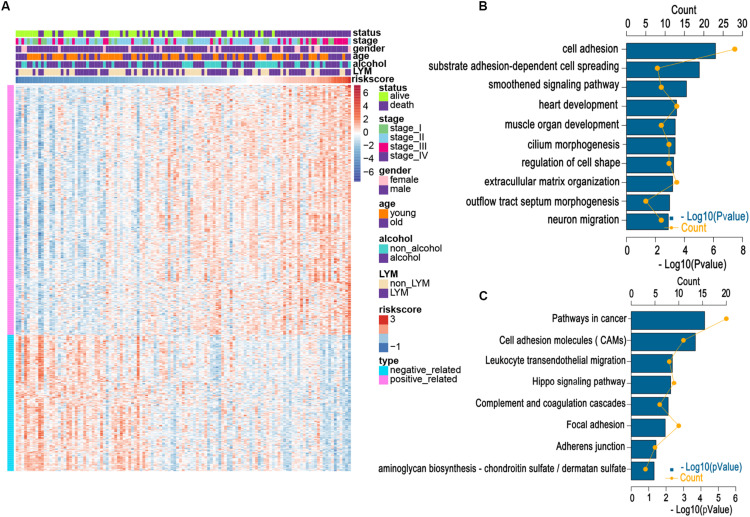
Relationship between risk score and most relevant immune-related genes and biological pathways. **(A)** Details of risk score and the most relevant genes. **(B)** Gene enrichment with GO terms of the selected genes. **(C)** Gene enrichment with KEGG terms of the selected genes. Young and old represent age <60 and age ≥60 years, respectively.

We applied a GO analysis to determine the biological roles of this signature. The genes with Pearson |*R*| > 0.4 were considered strongly linked to the risk score. We then generated a heatmap for these genes and the distribution of clinical features for every patient (Figure 4A). To identify fundamental biological functions, GO and KEGG analyses were carried out. We found that the risk score was related to several pathways, such as the cell adhesion, leukocyte transendothelial migration, and cancer progression, which may be associated with cancer metastasis (Figures 4B,C). These findings may indicate that patients in the high-risk group may be more likely to suffer lymphatic metastases.

### Relationship Between the Risk Score and Immune Landscapes

**FIGURE 5:**
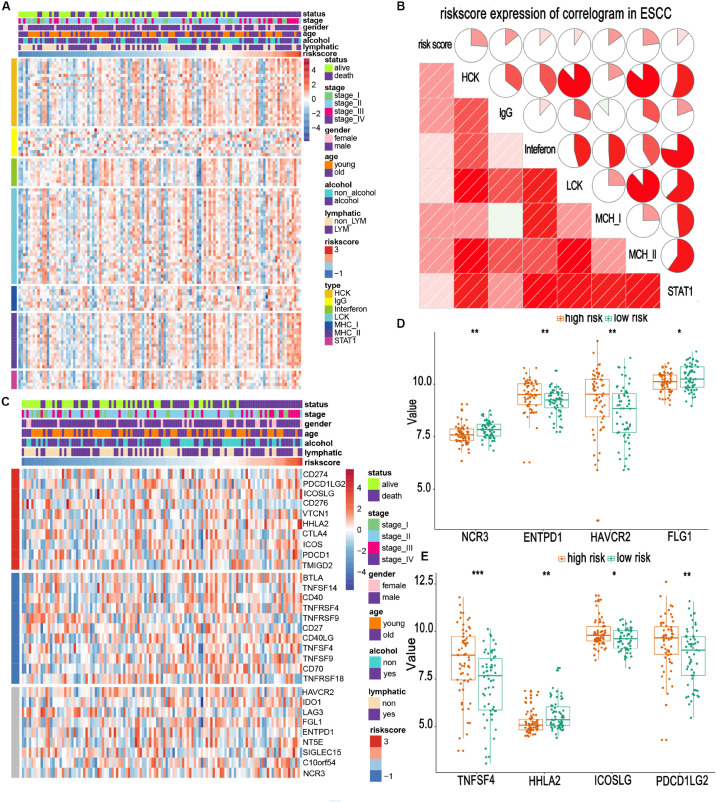
Relationship between risk scores and immune metagenes and immune checkpoints. **(A,C)** Expression of metagenes heatmap and corrgram in the training cohort. **(B)** The expression profile of immune checkpoints landscapes in the training cohort. **(D,E)** Different expression of immune checkpoints in high- and low-risk groups. *, **, and *** represent *P* < 0.05, *P* < 0.01, and *P* < 0.001, respectively. Young and old represent age <60 and age ≥60 years, respectively.

Owing to the establishment of the risk based on immune-related genes, we speculated that the risk might be relative to immune activities, immune response, and TME. First, we chose seven well-studied clusters of 104 genes in total, which were then defined as metagenes (HCK, interferon, LCK, MHC-I, MHC-II, STAT1), representing different types of inflammatory and immune responses. As illustrated in Figure 5A, we found that most clusters were positively associated with the risk score, such as HCK and MHC II clusters. These seven clusters were subjected to the GSVA to verify what we found in the seven metagene clusters. The results suggested that the high-risk score was mainly based upon genes related to the activation of macrophages and T-cell signaling transduction (Figure 5B).

Immune checkpoints are extremely essential molecules in the immune TME. Therefore, we sought to examine the correlation between the risk score and immune checkpoints expression. We altogether enrolled 30 immune checkpoints in our analysis, including TNF superfamily (*BTLA*, *TNFSF14*, *CD40*, *TNFRSF4*, *TNFRSF9*, *CD27*, *CD40LG*, *TNFSF4*, *TNFSF9*, *CD70*, and *TNFRSF18*) (Ward-Kavanagh et al., 2016), B7-CD28 family (*CD274*, *PDCD1LG2*, *ICOSLG*, *CD276*, *VTCN1*, *HHLA2*, *CTLA4*, *ICOS*, *PDCD1*, and *TMIGD2*) (Janakiram et al., 2015; Zhang et al., 2018, 2020), and other immune checkpoint members (*HAVCR2*, *IDO1*, *LAG3*, *FGL1*, *ENTPD1*, *NT5E*, *SIGLEC15*, *C10orf54*, and *NCR3*) (Chretien et al., 2019; Wang et al., 2019a, b). The heatmap for immune checkpoints expression was produced, taking other clinical characteristics into consideration, such as sex, age, TNM stage, and lymphatic metastasis (Figure 5C). We observed that *TNFSF4*, *ICOSLG*, *PDCD1LG2*, *HAVCR2*, and *ENTPD1* were obviously upregulated in patients of the high-risk group. In contrast, *HHLA2*, *NCR3*, and *FGL1* were downregulated (Figures 5D,E). Most of these upregulated molecules are potential targets for cancer immunotherapy (Du et al., 2017; Marinelli et al., 2018). This suggests that high-risk patients may benefit from the new immune targeted therapies.

**FIGURE 6:**
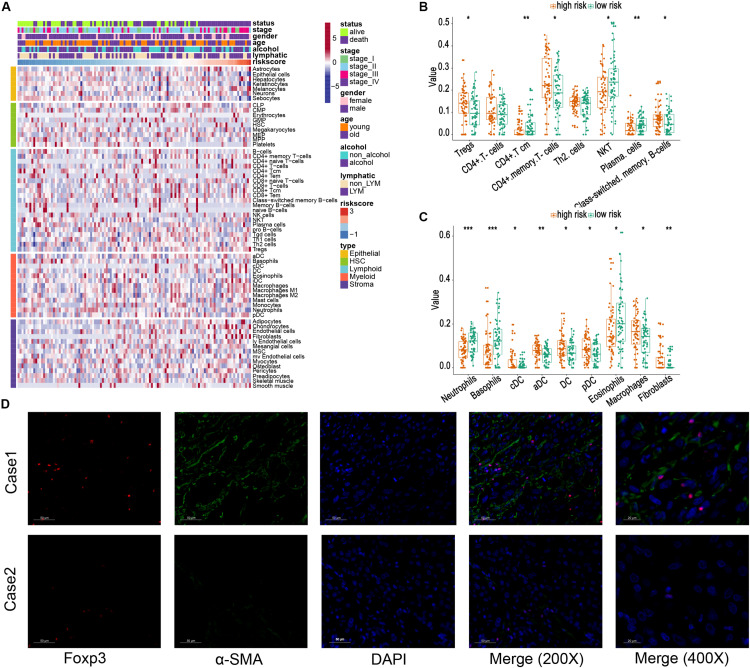
Relationship between risk score and different cells estimated by xCell. **(A)** The landscape of risk score and different cell infiltration. **(B,C)** Different distributions of estimated cells in high- and low-risk groups. **(D)** Immunofluorescence images of Tregs and fibroblasts in tissues from the high-risk group (Case 1) and low-risk group (Case 2), respectively. The foxp3 is marked as red, and α-SMA is marked as green (200×) *, **, and *** represent *P* < 0.05, *P* < 0.01, and *P* < 0.001, respectively. Young and old represent age <60 and age ≥60 years, respectively.

Then, the xCell method was performed to investigate the TME cell infiltration. According to the analyzed result, high-risk groups exhibited increased infiltration of regulatory T cells (Tregs), CD4^+^ memory T cells, memory B cells, macrophages, several dendritic cells, and fibroblasts, and low infiltration of plasma cells, neutrophils, basophils, eosinophils (Figures 6A–C). The previous research proved that Tregs could promote progression of ESCC, whereas both Tregs and fibroblasts were relevant to unfavorable survival in patients with ESCC (Fu et al., 2011; Nabeki et al., 2015; Yue et al., 2020). Meanwhile, α-SMA and Foxp3 are specific biomarkers of fibroblasts and Treg cells (Korn and Muschaweckh, 2019; Zhan et al., 2019). To confirm our analytical results, we first selected two representative tumor samples from the high- and low-risk groups and stained α-SMA and Foxp3 in the two tumor sample slices using the immunofluorescent assay method. The results, including case 1 (a low-risk patient) and case 2 (a high-risk patient), are shown in Figure 6D. These images verified that high-risk group patients demonstrate a higher infiltration of fibroblasts and Tregs.

### Popularized the Signature in the LUSC and Head and Neck Squamous Cell Carcinoma

**FIGURE 7:**
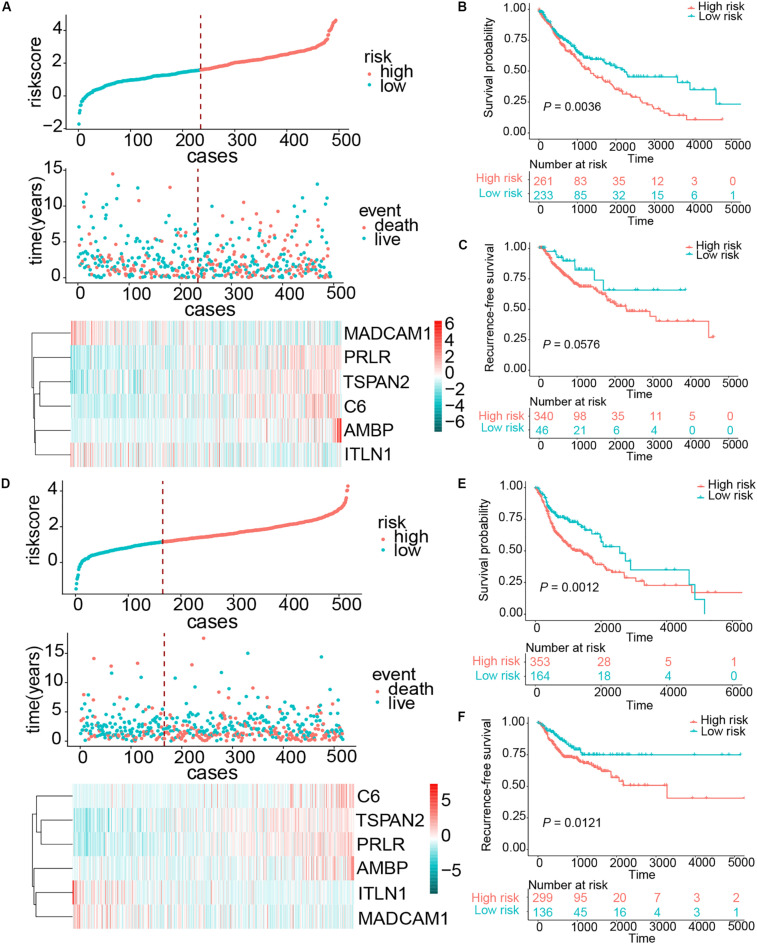
Risk score distribution and survival of patients in the LUSC and HNSCC cohorts. **(A)** The risk scores for 494 patients of the LUSC cohort, the survival of each patient, and gene expression distribution in the LUSC cohort. **(B,C)** Kaplan–Meier curve of OS and RFS in the LUSC cohort, respectively. **(D)** The risk scores for 517 patients of the HNSCC cohort, the survival of each patient, and gene expression distribution in the HNSCC cohort. **(E,F)** Kaplan–Meier curve of OS and RFS in the HNSCC cohort, respectively.

Esophageal squamous cell carcinoma resembled squamous carcinomas of other organs more than esophageal adenocarcinomas, especially LUSC and HNSC. Moreover, ESCC, LUSC, and head and neck squamous cell carcinoma (HNSCC) had familiar genetic backgrounds (Lin et al., 2018). We collected 494 LUSC cases and 517 HNSCC samples from the TCGA database. The same risk score formula was applied to these two cohorts. The patients were separated into high- and low-risk groups based on risk score. The patients with a high-risk score in the HNSCC cohort exhibited worse OS (HR = 1.6729, 95% CI = 1.1297–2.2936, *P* = 0.0012) and RFS (HR = 1.7928, 95% CI = 1.1293–2.8463, *P* = 0.0121). For the LUSC cohort, patients in the high-risk group also demonstrated worse OS (HR = 1.5045, 95% CI = 1.1407–21.9843, *P* = 0.0036); however, this risk score exhibited a borderline difference between high- and low-risk group in RFS (*P* = 0.0576) (Figure 7). These results suggest that our signature had a favorable promotion value.

## Discussion

Despite the combination of surgery, chemotherapy, and radiotherapy used to treat ESCC, patients continue to suffer poor clinical outcomes. Immunotherapy has produced promising clinical results for ESCC and is considered an emerging treatment for ESCC (Metges et al., 2019). Thus, it is essential to construct a useful and meaningful immune-related signature for ESCC. Such a signature could help us to assess the immune status of patients with ESCC. If applied correctly, this immune-related signature could act as a prognostic biomarker for ESCC and enable immunotherapeutic result stratification. Up to now, there was a limited immune-related signature to predict prognosis in patients with ESCC. We generated a six-gene–based immune-related signature through profiling an immune-related gene set. This signature exhibited a close connection with OS in patients with ESCC and effectively identified patients with ESCC at high risk of poor prognosis in the validation cohort. Meanwhile, high-risk patients exhibited an enhanced local immune phenotype in contrast to their low-risk counterparts. This indicates that high-risk cases may benefit from immunotherapies.

Our prognostic signature was constructed using various immune-related genes containing protective (*ITLN1* and *MADCAM1*) and risky (*TSPAN2*, *AMBP*, *C6*, and *PRLR*) genes. Regarding the two protective genes, *ITLN1* – also known as ometin-1 – is a 34-kDa secretory protein and pleiotropic adipocytokine, possessing metabolic, inflammatory, and immune-related properties. *ITLN1* is highly expressed in the visceral adipose tissue, particularly in the stromal vascular cell (Jaikanth et al., 2013). *ITLN1* also carries prognostic value for various malignant tumors, such as colorectal, gastric cancers, and neuroblastoma. *ITLN1* is a tumor suppressor in patients with gastric cancer and neuroblastoma. It is also related to improved survival in patients with gastric cancer (Li et al., 2015a, b). Kim et al. (2012) reported that *ITLN1* is a favorable prognostic biomarker in patients with stage IV colorectal cancer (CRC). In the contrary, a retrospective cohort research study revealed that there was a positive correlation between the level of circulating *ITLN1* concentrations and CRC risk (Aleksandrova et al., 2016). *MADCAM-1*, a principal ligand of α4 integrins, is mainly expressed on endothelial cells and high endothelial venules of gut-associated lymphoid tissues, mediating the recruitment and activation of lymphocytes (Rose et al., 2002; Kuhbandner et al., 2019). Steiniger et al. (2001) specialized that fibroblasts expressing *MAdCAM-1* may attract CD4^+^ T cells and instruct them into the periarteriolar T-cell area. However, there is no prognostic research of *ITLN1* and *MADCAM-1* in ESCC. Our findings suggest that *ITLN1* and *MADCAM1* are protective genes for ESCC. More studies are needed to further illuminate the prognostic value of *ITLN1* and *CAMDAM-1* and their relevant mechanisms in patients with ESCC.

The roles of *TPSNA2*, *AMBP*, and *PRLR* have not been confirmed in ESCC, but they are involved in the progression of various malignant cancers. For instance, *TPSNA2* performs a key function to suppress ROS production, leading to increased invasiveness and metastasis in lung and liver cancers. *TPSNA2* is also a poor outcome biomarker for patients with lung adenocarcinoma and a protective gene for patients with acute myeloid leukemia (Otsubo et al., 2014; Lin et al., 2020). *AMBP* is an important member of the lipocalin superfamily, modulating the processes of inflammation (Akerstrom et al., 2000). Sekikawa et al. pointed out that low expression of *AMBP* predicts an unfavorable prognosis in patients with oral squamous cell carcinoma (Sekikawa et al., 2018). *PRLP* acts as a vital receptor of PRL hormone. After their combination, the PRLP-PRL complex activates signals that suppress the epithelia–mesenchymal transition processes and promote the invasiveness of breast cancer cells (Nouhi et al., 2006). *PRLR* is an independent predictor of better outcomes in patients with breast cancer (Hachim et al., 2016). Conversely, it is a negative prognostic marker for patients with HNSCC (Bauernhofer et al., 2011). Finally, in our former study, *C6* was regarded as a risk-promoting factor in ESCC, in agreement with our present results (Li et al., 2017).

We further investigated the relevant and possible mechanisms of the local immune risk signature. The genes related to the risk score were predominantly focused on cell adhesion, leukocyte transendothelial migration, and cancer progression pathways, which are related to cancer metastasis. Subsequently, the relationship between the risk score and seven well-defined metagenes was explored (Rody et al., 2009). These seven clusters of metagenes represent the relatively comprehensive inflammation and immune response in the TME, including the functions of B lymphocytes (immunoglobulin G), macrophages and cells of the monocyte/myeloid lineage (HCK), T cells (LCK), major histocompatibility class II complex on professional antigen-presenting cells for their interaction with T cells (MHC-II), the major histocompatibility class I for the presentation of intracellular antigens (MHC-I), interferon signal transduction (STAT1), and the interferon response of cells (interferon). As a result, the risk score was found positively associated with HCK and MCH metagenes. Thus, high-risk scores were based on genes relevant to the activation of macrophages and T-cell signaling transduction. Interestingly, one of the key genes in the signature – *MADCAM1* – was closely related to immune cell infiltrations, especially for T cells (Mlecnik et al., 2010). Meanwhile, one of the risky genes – *TSPAN2* – inhibited macrophage secretion of lipopolysaccharide-induced tumor necrosis factor α (TNF-α) and interleukin 6 (IL-6) (Qiang et al., 2008). Tissue-resident macrophages expressing PRLR are able to promote fibrosis of the TME in cases of pancreatic cancer (Tandon et al., 2019). PRLR also influences the survival and differentiation of T-cell progenitors (Carreño et al., 2005). These observations further suggest that risk score is closely related to macrophage and T-cell activities. High-risk patients exhibited higher expression of *TNFSF4*, *ICOSLG*, *PDCD1LG2*, *HAVCR2*, and *ENTPD1.* These molecules are strongly associated with T-cell activation and responses. For example, *TNFSF4 –* also known as OX40L *–* is a ligand of OX40, and its combination with OX40 regulates T-cell proliferation, activation, and survival and even has an effect on cytokine release from T cells (Reuter et al., 2015). ICOS signaling helps regulate T_*H*_1, T_*H*_2, and T_*H*_17 immunity (Wikenheiser and Stumhofer, 2016). ICOSLG, a vital ligand of ICOS, also plays a crucial role in the regulation of T cell immunity (Marinelli et al., 2018). *PDCD1LG2*, *HAVCR2*, and *ENTPD1* contribute to immune tolerance in the TME and suppress the antitumor function of T cells (Sabatos et al., 2003; Rozali et al., 2012; Duhen et al., 2018). Considering that these immune checkpoints are potential and promising targets for cancer immunotherapies, the high expression of these molecules in the signature-based high-risk patients may provide an additional immunotherapeutic possibility.

We also found higher infiltration of Tregs in high-risk patients. Tregs are well-known mediators that contribute to immunologic tolerance, weakening T-cell activation, and responsiveness (Yang et al., 2019; Aykut et al., 2020). Tregs may also destabilize and reprogram under some certain acute proinflammatory signals such as IL-6 and interferon γ. This attenuates the immunosuppressive activities of Tregs, promoting a proinflammatory state and functioning as an antitumor agent (Munn et al., 2018). This emerging understanding may also provide a potential and prospective target for tumor immunotherapies. All these findings remind us that high-risk cases are more likely to profit from immunotherapies. In the meantime, these results can help us to comprehend the real immune status of patients within different risk cohorts, which also may be conductive to clinical instruction.

We constructed an initial immune-related signature to predict prognosis for patients with ESCC (Li et al., 2017); however, there were some limitations to this research. Although we enrolled approximately 708 immune-related genes, this may not be sufficient for a comprehensive analysis. On the other hand, our previous study lacked a qRT-PCR validation to avoid the false-positive error of sequencing. The design of this study addressed these limitations to some degree. We enrolled 2,630 immune-related genes in the present study, applied the robust risk score method, and further validated our findings in an independent cohort. However, our present study also has several limitations. This project lacked a large population cohort to make further validations. Additionally, this was retrospective research and should be tested in prospective cohorts. Finally, tumors are considered heterogenic tissues, especially for the immune TME. Considering that a tumor’s characteristics and composition vary by location, the predictive capacity of our six-gene immune-related signature may vary in different areas with tumor tissue.

In conclusion, we established a feasible and reproducible immune-related risk signature for ESCC and furnished new information related to immune profiling of ESCC. The clinical value and application range of this signature cannot be ignored. We also believe our findings may assist clinicians decide on individual management and treatment strategies for patients with ESCC.

## Data Availability Statement

The datasets presented in this study can be found in online repositories. The names of the repository/repositories and accession number(s) can be found in the article/Supplementary Material.

## Ethics Statement

This research was approved by the Ethics Committee Board of The First Affiliated Hospital of Zhengzhou University.

## Author Contributions

JH and NS designed the study. CZ performed the analysis. CZ and YL wrote the manuscript and contributed to the immunofluorescence of clinical samples. ZheZ and YZ performed the validation in the independent cohort. ZhiZ, GZ, FW, and LF developed the inclusion criteria and normalized the expression profile data. YC contributed to preparing the figures and tables. All authors contributed to the article and approved the submitted version.

## Conflict of Interest

The authors declare that the research was conducted in the absence of any commercial or financial relationships that could be construed as a potential conflict of interest.

## Footnotes

1 https://cancergenome.nih.gov/

2 http://david.abcc.ncifcrf.gov/home.jsp

3 https://cytoscape.org/

4 http://xCell.ucsf.edu/

5 https://www.r-project.org

## Supplementary Material

Supplementary Figure 1 The six-immune-related gene signature generation pipeline.

Supplementary Figure 2 Survival analysis of all ESCC patients in the training cohort stratified by lymphatic metastasis and stage. **(A,B)** Kaplan-Meier curves of OS in LN+ and LN− patients of the training cohort. **(C,D)** Kaplan-Meier curves of OS in early and advanced stages.

Supplementary Figure 3 Survival analysis of all ESCC patients in the training cohort stratified by age and gender. **(A,B)** Kaplan-Meier curves of OS in older and younger patients of the training cohort. **(C,D)** Kaplan-Meier curves of OS in male and female patients of the training cohort.

Supplementary Figure 4 Survival analysis of all ESCC patients in the training cohort stratified by tobacco and alcohol history. **(A,B)** Kaplan-Meier curves of OS in smoker and non-smoker patients of the training cohort. **(C,D)** Kaplan-Meier curves of OS in drinker and non-drinker patients of the training cohort.

## Abbreviations

AUC: Area under the curve
CI: Confidence interval
ESCC: Esophageal squamous cell carcinoma
GO: Gene Oncology
GVSA: Gene Set Variation Analysis
HNSCC: Head and neck squamous cell carcinoma
HR: Hazard ratio
KEGG: Kyoto Encyclopedia of Genes and Genomes
LUSC: Lung squamous cell carcinoma
NA: Not available
OS: Overall survival
qRT-PCR: Quantitative real-time polymerase chain reaction
RFS: Recurrence-free survival
ROC: Receiver operating characteristic
TME: Tumor environment.
