# Supplementary figures and images for: Identification of a Prognostic Immune Signature for Esophageal Squamous Cell Carcinoma to Predict Survival and Inflammatory Landscapes
Source: Front Cell Dev Biol. 2020 Dec 17;8:580005. doi: 10.3389/fcell.2020.580005 (PMC7773787; doi:10.3389/fcell.2020.580005)

## Supplementary Figure 1

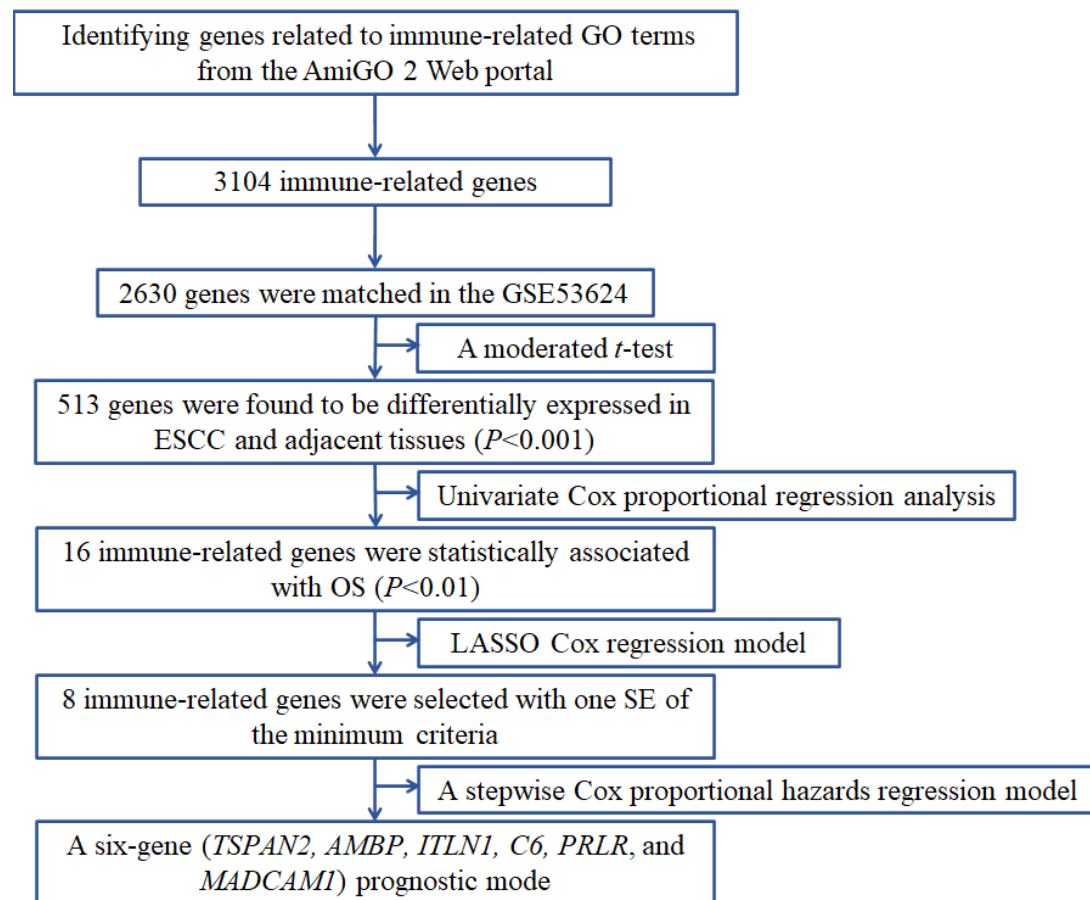

Supplementary Figure 2

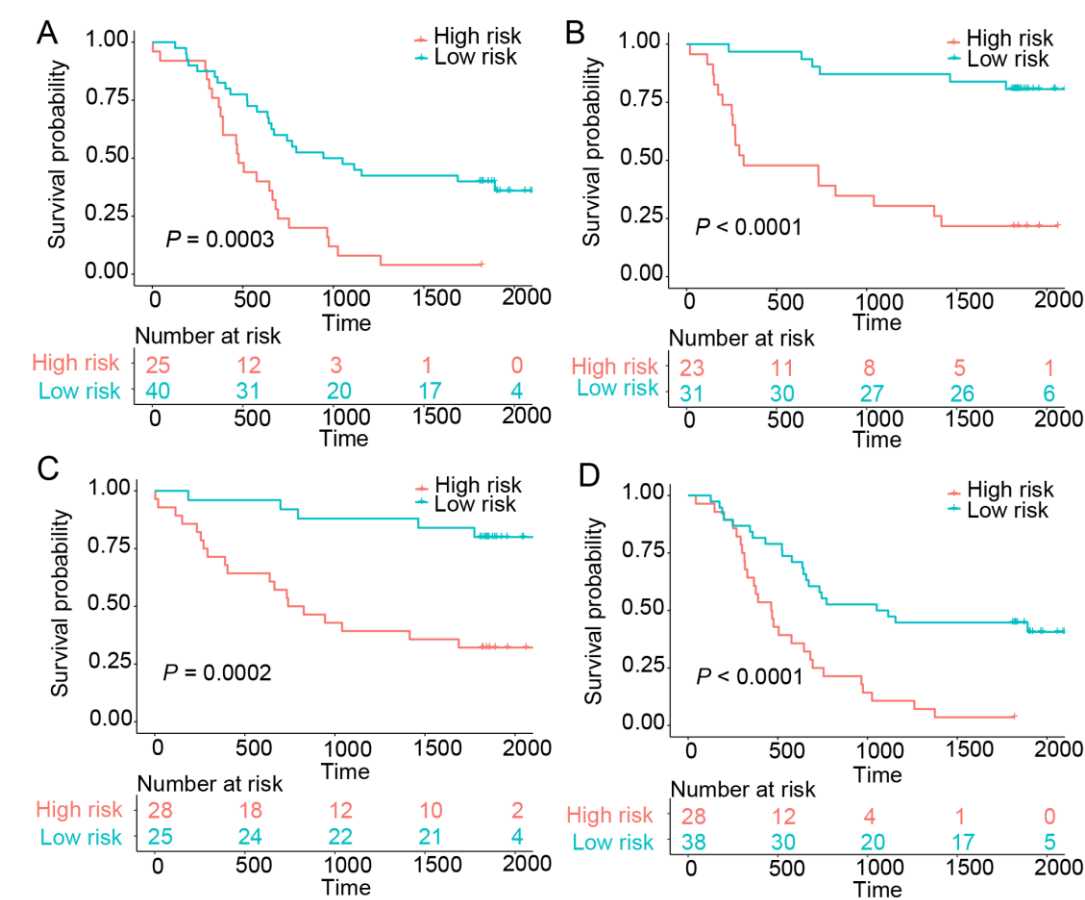

Supplementary Figure 3

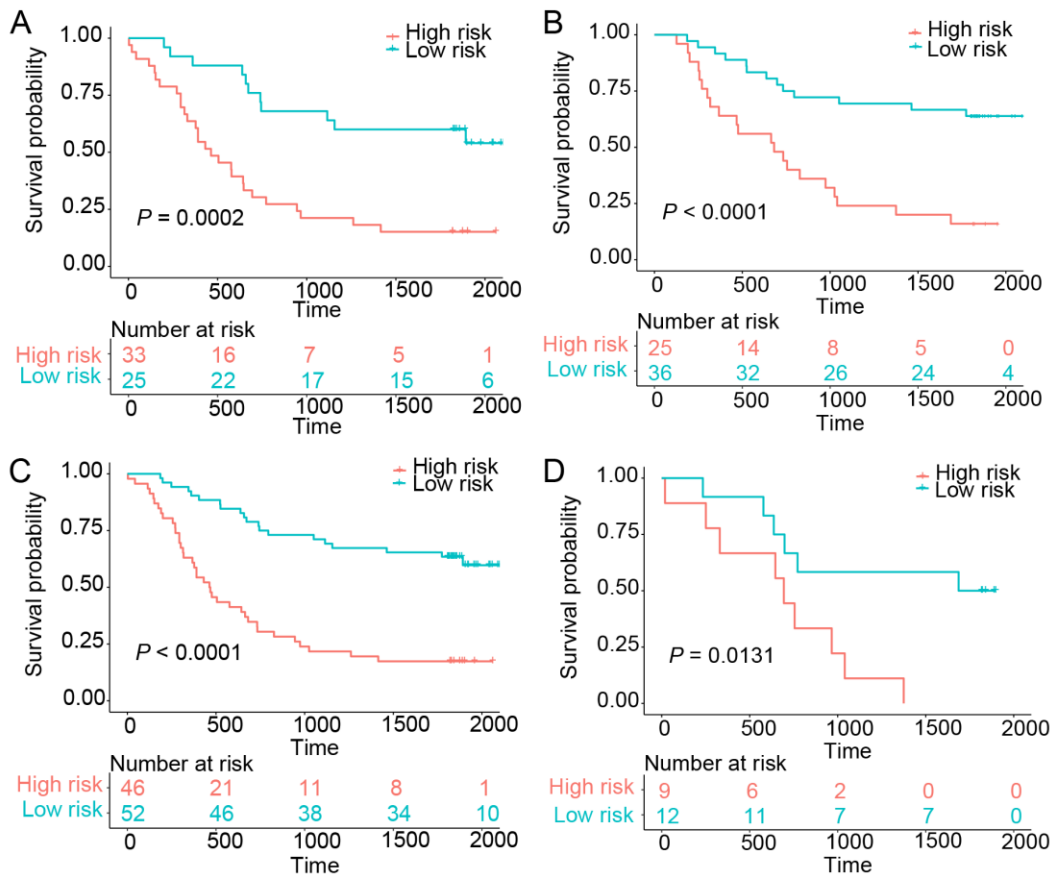

Supplementary Figure 4

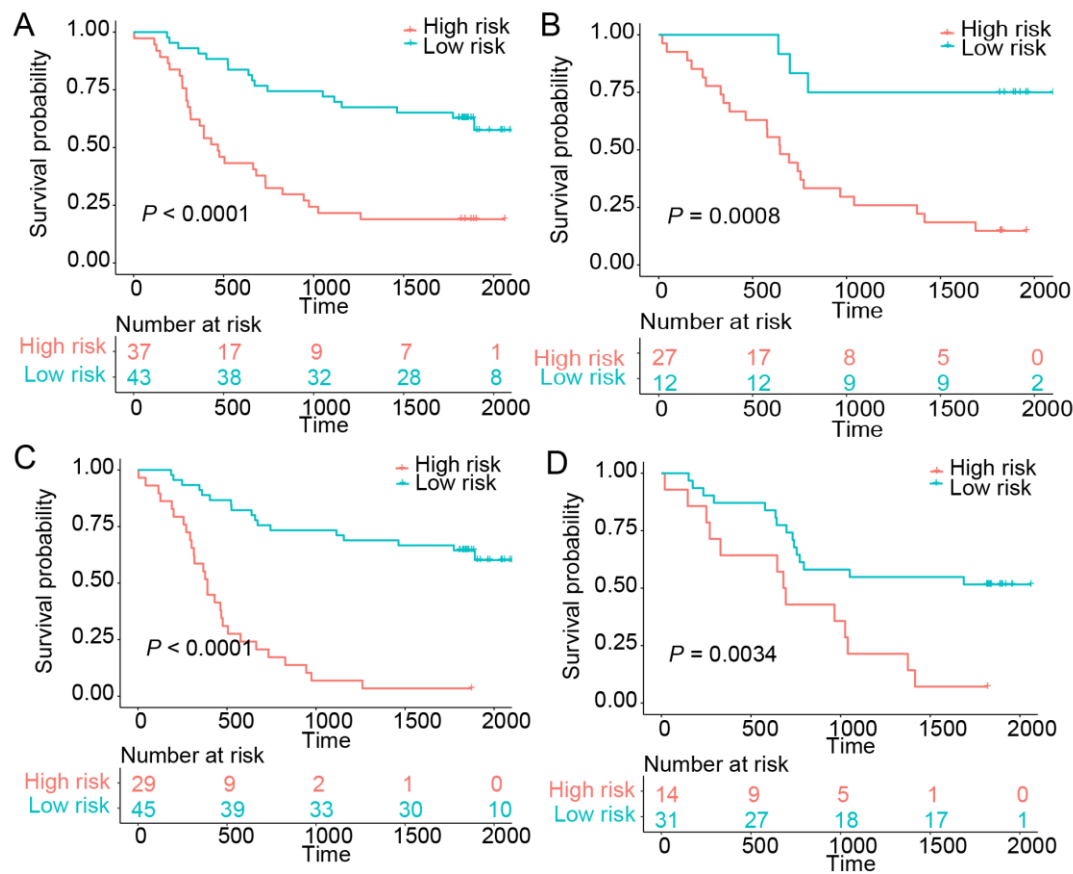

Supplement: Supplementary Figure 1 — The six-immune-related gene signature generation pipeline. [file Data_Sheet_1.PDF]
