# Supplementary material for: Identification of a Prognostic Immune Signature for Esophageal Squamous Cell Carcinoma to Predict Survival and Inflammatory Landscapes
Source: Front Cell Dev Biol. 2020 Dec 17;8:580005. doi: 10.3389/fcell.2020.580005 (PMC7773787; doi:10.3389/fcell.2020.580005)
Supplement: Supplementary file 2 [file Table_1.docx]

Supplementary table 1. Primer Sequences for q-PCR.

| Gene Name | Forward Primer | Reverse Primer |
| --- | --- | --- |
| *TSPAN2* | 5'-TGTGGGGCTGTATGTTCTGG-3' | 5'-AGCACACATTGCGACTCCC-3' |
| *AMBP* | 5'-ATGGACCCACCATTACTGCC-3' | 5'-CCAGGGACACATTCACCTCG-3' |
| *ITLN1* | 5'-ACGTGCCCAATAAGTCCCC-3' | 5'-CCGTTGTCAGTCCAACACTTTC-3' |
| *C6* | 5'-TTGATGGGCAATGGGTTTCAT-3' | 5'-ACTTGTCCTACTGCTTTTGACAG-3' |
| *PRLR* | 5'-TCTCCACCTACCCTGATTGAC-3' | 5'-CGAACCTGGACAAGGTATTTCTG-3' |
| *MADCAM1* | 5'-GGGAGAAGTGATCCCAACAGG-3' | 5'-CGTTTCCAGAGGTGATACGTG-3' |
| *GAPDH* | 5'-TCCAAGGATTGTGGTTATGGAGA-3' | 5'-AGCACAGGTGATGCAACTCTG-3' |

Supplementary table 2. Clinical characteristics of the patients from multiple institutions.

| Characteristics | GSE53624  cohort  N=119 | Independent  cohort  N=77 |
| --- | --- | --- |
| Age, year |  |  |
| ≥60 | 58 | 56 |
| <60 | 61 | 21 |
| Sex |  |  |
| Male | 98 | 58 |
| Female | 21 | 19 |
| Smoking history |  |  |
| Yes | 80 | NA |
| No | 39 | NA |
| Alcohol history |  |  |
| Yes | 74 | NA |
| No | 45 | NA |
| Tumor grade |  |  |
| well | 23 | NA |
| moderate | 64 | NA |
| poor | 32 | NA |
| Tumor location |  |  |
| Upper | 14 | NA |
| Middle | 69 | NA |
| Lower | 36 | NA |
| Lymphatic metastasis |  |  |
| Yes | 65 | 35 |
| No | 54 | 42 |
| TNM stage |  |  |
| I and II | 53 | 51 |
| III and Ⅳ | 66 | 26 |
| OS state |  |  |
| Alive | 46 | 53 |
| Death | 73 | 24 |

NA, not available; OS, overall survival.

Supplementary table 3. Univariate Cox proportional regression analysis of the valued prognostic immune-related genes in GSE53624 cohort.

Abbreviations: HR, hazard ratio; CI, confidence interval.

|  | Univariable analysis | | |
| --- | --- | --- | --- |
| Gene symbol | HR | 95%CI | *P* value |
| *TSPAN2* | 1.3283 | 1.1336-1.5564 | 0.0004 |
| *AMBP* | 1.2844 | 1.1098-1.4865 | 0.0008 |
| *ITLN1* | 0.7197 | 0.5924-0.8743 | 0.0009 |
| *C6* | 1.5438 | 1.1629-2.0495 | 0.0027 |
| *PRLR* | 1.2019 | 1.0609-1.3617 | 0.0039 |
| *ITGA9* | 1.3674 | 1.1021-1.6964 | 0.0045 |
| *RBM47* | 0.5970 | 0.4176-0.8534 | 0.0047 |
| *PGLYRP3* | 0.8365 | 0.7387-0.9474 | 0.0049 |
| *HMGB4* | 0.7557 | 0.6209-0.9196 | 0.0052 |
| *FOXL1* | 1.2428 | 1.0660-1.4489 | 0.0055 |
| *PDE5A* | 1.5139 | 1.1274-2.0329 | 0.0058 |
| *PLAU* | 1.5268 | 1.1280-2.0665 | 0.0061 |
| *PGM2* | 0.5998 | 0.4138-0.8695 | 0.0070 |
| *IL18* | 0.7257 | 0.5745-0.9167 | 0.0072 |
| *PLA2G3* | 0.8608 | 0.7710-0.9611 | 0.0077 |
| *MADCAM1* | 0.7228 | 0.5683-0.9192 | 0.0081 |

|  | Univariable analysis | | |  | Multivariable analysis | | |
| --- | --- | --- | --- | --- | --- | --- | --- |
| Variable | HR | 95%CI | *P* value |  | HR | 95%CI | *P* value |
| Age |  |  |  |  |  |  |  |
| ≥60 or <60 | 0.7335 | 0.4276-1.2583 | 0.2604 |  |  |  |  |
| Sex |  |  |  |  |  |  |  |
| Male or female | 0.8516 | 0.4166-1.7409 | 0.6598 |  |  |  |  |
| Smoking history |  |  |  |  |  |  |  |
| Yes or no | 0.9911 | 0.5642-1.7409 | 0.9751 |  |  |  |  |
| Alcohol history  Yes or no  Tumor location | 1.1807 | 0.6813-2.0462 | 0.5538 |  |  |  |  |
| Upper, middle or lower | 1.6179 | 0.8527-3.0700 | 0.1410 |  |  |  |  |
| Tumor grade |  |  |  |  |  |  |  |
| Well, moderate or poor | 1.0466 | 0.6901-1.6874 | 0.8302 |  |  |  |  |
| T stage |  |  |  |  |  |  |  |
| 1, 2, 3 or 4 | 1.4561 | 0.9995-2.1213 | 0.0503 |  |  |  |  |
| Lymphatic metastasis |  |  |  |  |  |  |  |
| Yes or no | 2.6628 | 1.4839-4.7785 | 0.0010 |  | 1.6991 | 0.6466-4.4649 | 0.2822 |
| TNM stage |  |  |  |  |  |  |  |
| I, II or III | 2.9056 | 1.6608-5.0835 | 0.0002 |  | 2.0263 | 0.7215-5.6909 | 0.1801 |
| Risk score |  |  |  |  |  |  |  |
| High or low | 2.2670 | 1.3143-3.9104 | 0.0033 |  | 2.1854 | 1.1756-0.0135 | 0.0135 |

Supplementary table 4. Univariable and multivariable Cox regression analysis of the six-gene immune-related signature and recurrence-free survival in GSE53624 cohort.

Abbreviations: HR, hazard ratio; CI, confidence interval.

Supplementary table 5. Univariable and multivariable Cox regression analysis of the six-gene immune-related signature and recurrence-free survival in the independent validation cohort.

|  | Univariable analysis | | |  | Multivariable analysis | | |
| --- | --- | --- | --- | --- | --- | --- | --- |
| Variable | HR | 95%CI | *P* value |  | HR | 95%CI | *P* value |
| Age |  |  |  |  |  |  |  |
| ≥60 or <60 | 1.2962 | 0.4656-3.6085 | 0.6195 |  |  |  |  |
| Sex |  |  |  |  |  |  |  |
| Male or female | 0.4955 | 0.1940-1.2654 | 0.1421 |  |  |  |  |
| Lymphatic metastasis |  |  |  |  |  |  |  |
| Yes or no | 15.0863 | 3.4731-65.5308 | 0.0003 |  | 12.6631 | 2.4383-65.7643 | 0.0025 |
| TNM stage |  |  |  |  |  |  |  |
| I, II or III | 2.8379 | 1.6587-4.8552 | 0.0001 |  | 1.0207 | 0.4172-2.4973 | 0.9641 |
| Risk score |  |  |  |  |  |  |  |
| High or low | 4.7875 | 1.8440-12.4295 | 0.0013 |  | 3.6539 | 1.1205-11.9149 | 0.0316 |

Abbreviations: HR, hazard ratio; CI, confidence interval.
